# FHL2 promotes tubular epithelial‐to‐mesenchymal transition through modulating β‐catenin signalling

**DOI:** 10.1111/jcmm.13446

**Published:** 2017-11-29

**Authors:** Ting Cai, Danqin Sun, Ying Duan, Yumei Qiu, Chunsun Dai, Junwei Yang, Weichun He

**Affiliations:** ^1^ Center for Kidney Disease Second Affiliated Hospital Nanjing Medical University Nanjing Jiangsu China

**Keywords:** FHL2, β‐catenin, TGF‐β1, EMT, tubular epithelial cells, renal fibrosis

## Abstract

β‐Catenin signalling plays an important role in regulating tubular epithelial‐to‐mesenchymal transition (EMT), an indispensable programme for driving renal fibrosis. As an adapter protein, four and a half LIM domain protein 2 (FHL2) acts as a coregulator of β‐catenin in several other cell types. To determine whether FHL2 affects β‐catenin signalling and thus is involved in tubular EMT, we examined its expression and function in the process of TGF‐β1‐induced EMT. FHL2 mRNA and protein were induced by TGF‐β1 in rat tubular epithelial cells (NRK‐52E), an effect that intracellular Smad signalling was required. Ectopic expression of FHL2 inhibited E‐cadherin and enhanced α‐smooth muscle actin (α‐SMA) and fibronectin expression, whereas knockdown of FHL2 partially restored E‐cadherin and reduced α‐SMA and fibronectin induction stimulated by TGF‐β1. Overexpression of FHL2 increased β‐catenin dephosphorylation (Ser37/Thr41), nuclear translocation and β‐catenin‐mediated transcription and up‐regulated expression of β‐catenin target, EMT‐related genes, such as Snail, Twist, vimentin, plasminogen activator inhibitor‐1 and matrix metalloproteinase‐7. Conversely, knockdown of FHL2 increased β‐catenin phosphorylation (Ser33/37/Thr41), decreased its nuclear translocation and inhibited β‐catenin‐mediated transcription and target genes expression. TGF‐β1 induced a FHL2/β‐catenin interaction in NRK‐52E cells, especially in the nuclei. In a mouse model of obstructive nephropathy, FHL2 mRNA and protein were induced in a time‐dependent fashion, and the extent and pattern of renal β‐catenin activation were positively correlated with FHL2 induction. Collectively, this study suggests that FHL2, *via* modulating β‐catenin signalling, may implicate in regulation of TGF‐β1‐mediated tubular EMT and could be a potential therapeutic target for fibrotic kidney disease.

## Introduction

More than 10% of the population worldwide suffers from chronic kidney disease (CKD), and end‐stage renal failure is an ineludible outcome in a considerable proportion of patients with CKD 1, 2. Renal fibrosis, a major pathological manifestation in CKD, is characterized by relentless accumulation of abundant extracellular matrix (ECM) in the interstitial space, leading to replacement of normal tissue by scar tissue and widespread demolition of renal parenchyma 3, 4. Hence, a better understanding of the pathogenesis of renal fibrosis, especially the pivotal signal pathways involved, is momentous and necessary for exploiting efficacious strategies to retard, even arrest, this devastating pathologic process.

As myofibroblast is well known as the main type of ECM‐producing cells in the interstitium of fibrotic kidney, the origin of myofibroblasts during renal fibrosis has been intensely investigated and debated for several years 5. One of the controversies is the relative contribution of injured tubular epithelial cells going through epithelial‐to‐mesenchymal transition (EMT) to the myofibroblast population 6. By genetic cell lineage tracing, studies have suggested that tubular EMT, a phenotypic conversion programme in which the cells lose their epithelial markers and obtain mesenchymal properties, could not directly contribute to a substantial proportion of interstitial myofibroblasts in fibrotic kidney 5, 7. However, recent studies demonstrate that the tubular EMT is an essential and necessary procedure for driving renal fibrosis development 8, 9 and that inhibiting tubular EMT could be an efficient strategy for alleviating the progression of fibrotic CKD 3, 8, 9, 10.

Previous studies present that activation of β‐catenin signalling induces tubular EMT and enhances the magnitude of EMT induced by TGF‐β1 11, 12. As a principal mediator of canonical Wnt signalling pathway, β‐catenin signal cascade plays an important role in governing organ development, tissue homoeostasis and pathologic process of various human disorders 13. Once initiated by its upstream activator, β‐catenin is stabilized, rendering it to translocate into the nucleus, where it binds to T cell factor (TCF)/lymphoid enhancer‐binding factor (LEF) to trigger the transcription of β‐catenin target genes 14. Despite comparative quiescent in normal adult kidney, Wnt/β‐catenin signalling is definitely reactivated in various fibrotic kidney diseases 15. In tubular epithelial cells, activation of this signalling brings about the up‐regulation of numerous target genes associated with EMT and fibrogenesis, such as Snail1, matrix metalloproteinase‐7 (MMP‐7), plasminogen activator inhibitor‐1 (PAI‐1) and fibronectin 11, 16, 17. Apart from Wnts, several other pathogenic cues, for example integrin‐linked kinase, TGF‐β1 and angiotensin II, also regulate activation of β‐catenin signalling in fibrotic kidney 15. So, β‐catenin signalling could be a converging effector of multiple fundamental fibrogenic signal pathways. Accordingly, either inhibiting β‐catenin transcription activity or antagonizing its upstream Wnt is able to inhibit renal fibrosis 11, 18. Therefore, it is conceivable that repressing the activity of β‐catenin signalling could become an effective method to hinder the progression of fibrotic CKD.

It has been proposed that four and a half LIM domain‐only protein 2 (FHL2) is implicated in Wnt/β‐catenin signalling pathway 19. Protein containing Linl‐1, Isl‐1 and Mec‐3 (LIM) domains instinctively exhibits the property of protein–protein interaction because a LIM domain is constructed from two zinc finger domains that are separated by two amino acids, which builds a compact configuration with flexible connector structures on both sides 20, 21. FHL2 is a member of FHL subfamily belonging to LIM‐only proteins family. Based on its structures, FHL2 primarily functions as an adaptor or scaffold protein and is capable of interacting with manifold intracellular protein partners including β‐catenin 22. According to existing reports, the major mission of FHL2 is to modulate intracellular signalling pathways involved in various cellular processes 22. One recent study demonstrates that, as a coactivator of Wnt/β‐catenin signalling, FHL2 promotes podocyte damage and proteinuria in diabetic kidney disease (DKD) 23. However, it is hardly known about the expression and function of FHL2 in tubular epithelial cells in both physiologic and pathologic conditions. And the regulatory correlation between FHL2 and β‐catenin and its potential role in tubular EMT and renal fibrosis remain unknown too.

The data in this study indicate that expression of FHL2 is up‐regulated in the tubular epithelial cells treated with TGF‐β1 and in the fibrotic kidney after obstructive injury. And the further investigations reveal that FHL2 functionally implicates in regulating tubular EMT, and it functions, at least partially, through modulating the activity of β‐catenin signalling.

## Materials and methods

### Cell culture and treatment

Rat proximal tubular epithelial cell (NRK‐52E) was provided by the Cell Resource Center of the Shanghai Institutes for Biological Sciences, Chinese Academy of Sciences (Shanghai, China). The cells were cultured in Dulbecco's modified Eagle's medium–Ham's F12 medium supplemented with 10% foetal bovine serum (Gibco, Grand Island, NY). Serum‐starved cells were incubated with recombinant TGF‐β1 (R&D Systems, Minneapolis, MN, USA) for different periods of time at the concentration of 2 ng/ml except as otherwise indicated. The cells were then harvested for real‐time RT‐PCR and Western blot analyses. In some experiments, cells were pre‐treated with several chemical inhibitors at the concentrations specified for 30 min., followed by incubating with or without 2 ng/ml TGF‐β1. PD98059 (MEK1 inhibitor) and Wortmannin (PI3K inhibitor) were provided by Sigma‐Aldrich (Saint Louis, MO, USA), SP600125 (JNK inhibitor) was provided by Merck (Germany), and SB202190 (p38 MAPK inhibitor) and SB431542 (TGF‐β1 type I receptor inhibitor) were provided by R&D Systems.

### Plasmid transient transfection

Plasmid transient transfection was performed as described previously 24. NRK‐52E cells were transfected with DDK‐tagged FHL2 expression vector (RR207061; OriGene, Rockville, MD, USA) or Smad7 expression vector (Invitrogen, Carlsbad, CA) for 48 hrs. The empty vector pcDNA3 (Invitrogen) was used as a mock transfection control.

### Small interfering RNA inhibition

NRK‐52E cells were transiently transfected with negative control small interfering RNA (siRNA) or FHL2 siRNA (GenePharma, Shanghai, China) for 48 hrs using Lipofectamine 2000 reagent according to the manufacturer's instructions as described previously 25. In some experiments, cells were transfected with siRNA for 24 hrs, followed by incubating with or without 2 ng/ml of TGF‐β1 for different periods of time as indicated.

### RT‐PCR and real‐time RT‐PCR

Total RNA was extracted using TRIzol RNA isolation system (Invitrogen). The first strand of cDNA was synthesized using 2 μg of RNA in 20 μl of reaction buffer by reverse transcription using ReverTra Ace (Toyobo, Osaka, Japan) and oligo(dT)12–18 primers at 42°C for 30 min. 25. PCR was performed using a standard PCR kit with a 1 μl aliquot of cDNA and HotStarTaq polymerase (Qiagen, Valencia, CA, USA) with specific primer pairs 24. For quantitative determination of mRNA levels, a real‐time RT‐PCR was performed on an ABI PRISM 7300 Sequence Detection System (Applied Biosystems, Foster City, CA, USA), as described previously 25. The PCR reaction mixture in a 25 μl volume contained 12.5 μl of 2 ×  SYBR Green PCR Master Mix (Applied Biosystems), 5 μl of diluted reverse transcriptase product (1:10) and 0.5 μmol/l primer sets. The mRNA levels of detected genes were calculated after normalizing with GAPDH or β‐actin. The sequences of the primer pairs were as follows: FHL2 (rat) forward 5′‐CACCGACTGCTATTCCAACG‐3′ and reverse 5′‐GTGAAGCGTTGCCCAGATAG‐3′; FHL2 (mouse) forward 5′‐AGACCTGCTTCACCTGTCAG‐3′ and reverse 5′‐GTGAAGCGTTGCCCAGATAG‐3′; Smad7 forward 5′‐CCATCAAGGCTTTTGACTATGAAA‐3′ and reverse 5′‐CGGTGAAGCCCGTCCAT‐3′; β‐actin forward 5′‐CAGCTGAGAGGGAAATCGTG‐3′ and reverse 5′‐CGTTGCCAATAGTGATGACC‐3′; GAPDH forward 5′‐CAGCAAGGATACTGAGAGCAAGAG‐3′ and reverse 5′‐GGATGGAATTGTGAGGGAGATG‐3′.

### Western blot analysis

Cell lysates and kidney tissue homogenate were prepared, and Western blot analysis of protein expression was performed using routine procedures as described previously 26. The primary antibodies were used as follows: anti‐FHL2 (K0055‐3; Medical & Biological Laboratories, Japan), anti‐DDK (TA50011; OriGene), anti‐E‐cadherin (#610181; BD Transduction, San Jose, CA, USA), antifibronectin (F3648; Sigma‐Aldrich), anti‐α‐smooth muscle actin (α‐SMA) (A5228; Sigma‐Aldrich), anti‐β‐catenin (#610154; BD Transduction), anti‐dephosphorylated active‐β‐catenin (#05‐665; Millipore, Bellerica, MA, USA), anti‐phospho‐β‐catenin (Ser33/37/Thr41) (#9561; Cell Signaling, Boston, MA, USA), anti‐Snail (ab17732; Abcam, Cambridge, MA, USA), anti‐Twist (sc‐15393; Santa Cruz Biotechnology, Dallas, TX, USA), anti‐vimentin (#3932S; Cell Signaling), anti‐PAI‐1 (sc‐8979; Santa Cruz), anti‐MMP‐7 (GTX104658; GeneTex, Irvine, CA, USA), anti‐TBP (ab818; Abcam), anti‐GAPDH (sc‐25778; Santa Cruz Biotechnology) and anti‐α‐tubulin (sc‐53646; Santa Cruz Biotechnology).

### Nuclear and cytoplasmic fractionation

For preparation of nuclear protein, NRK‐52E cells were collected and washed twice with cold PBS. Nuclear protein was extracted using NE‐PER Nuclear and Cytoplasmic Extraction Reagents, on the basis of the protocols provided by the manufacturer (Thermo Scientific, Rockford, IL, USA), as described previously 27.

### Coimmunoprecipitation

Immunoprecipitation was performed using an established method 27. Briefly, NRK‐52E cells were lysed on ice in 1 ml of nondenaturing lysis buffer that contained 1% Triton X‐100, 0.01 M Tris‐HCl (pH 8.0), 0.14 M NaCl, 0.025% NaN3, 1% protease inhibitors cocktail and 1% phosphatase inhibitors cocktail I and II (Sigma‐Aldrich). After pre‐clearing with normal IgG, cell lysates (0.5 mg of protein) were incubated with 4 μg of anti‐β‐catenin (BD Transduction) or anti‐FHL2 (sc‐13409; Santa Cruz) at 4°C overnight, followed by precipitation with 30 μl of protein A/G Plus‐agarose for 1 hr at 4°C. The precipitated complexes were analysed by Western blotting with anti‐FHL2 or anti‐β‐catenin antibody. In some experiments, the nuclei extracted from cells were lysed and analysed by immunoprecipitation approach.

### Immunofluorescence staining

Immunofluorescence staining of cultured NRK‐52E cells was carried out using a routine protocol 11. Briefly, cells seeded on coverslips were fixed with methanol and acetone (1:1) for 10 min. at −20°C, blocked with 20% normal donkey serum for 30 min. at room temperature and then incubated with primary antibodies against E‐cadherin, fibronectin, α‐SMA, FHL2 or β‐catenin. For visualizing the primary antibodies, cells were stained with cyanine Cy3‐ or Cy2‐ conjugated secondary antibody (Sigma‐Aldrich). Cells were double stained with 4′, 6‐diamidino‐2‐phenylindole‐HCl (DAPI) to visualize the nuclei. Cells were viewed under a Nikon Eclipse 80i Epi‐fluorescence microscope.

### Immunohistochemical staining

Immunohistochemical staining of paraffin‐embedded mouse kidney sections (3 μm thickness) was carried out by an established procedure as described previously 18. The sections were stained with anti‐FHL2 antibody using the Vector M.O.M immunodetection kit, according to the instructions of manufacturer (Vector Laboratories, Burlingame, CA, USA). Slides were viewed under a Nikon Eclipse 80i microscope equipped with a digital camera (DS‐Ri1, Nikon, Shanghai, China).

### Transfection and luciferase assay

The effect of FHL2 on β‐catenin‐mediated transcription was determined using the TOPflash TCF reporter plasmid containing two sets of three copies of the TCF binding site upstream of the thymidine kinase (TK) minimal promoter and luciferase open reading frame (Millipore). NRK‐52E cells were cotransfected with TOPflash plasmid (1 μg) and either FHL2 expression vector or empty vector pcDNA3. For normalizing the transfection efficiency, an internal control reporter plasmid (0.1 μg) *Renilla reniformis* luciferase driven under TK promoter (pRL‐TK; Promega, Madison, WI) was also cotransfected. Luciferase assay was carried out by utilizing a dual luciferase assay system kit according to the protocol specified by manufacturer (Promega). Relative luciferase activity (arbitrary units) was shown by the fold induction over the controls.

### Animal model

Male CD‐1 mice weighing 20–22 g were provided by the Shanghai Experimental Animal Center (Shanghai, China). Unilateral ureteral occlusion (UUO) was performed using an established protocol, as described previously 18. Sham‐operated mice were used as normal controls. Mice were killed at various time‐points as indicated after UUO, and kidneys were removed for various analyses. The animal protocols were approved by the Institutional Animal Care and Use Committee at Nanjing Medical University.

### Statistical analysis

All data examined were presented as mean ± S.E.M. Statistical analysis of the data was processed by SigmaStat software (Jandel Scientific Software, SanRafael, CA, USA). Comparisons between groups were made using one‐way analysis of variance, followed by the Student–Newman–Keul's test. A *P* value ˂ 0.05 was considered statistically significant.

## Results

### TGF‐β1 induces FHL2 expression in tubular epithelial cells

**Figure 1:**
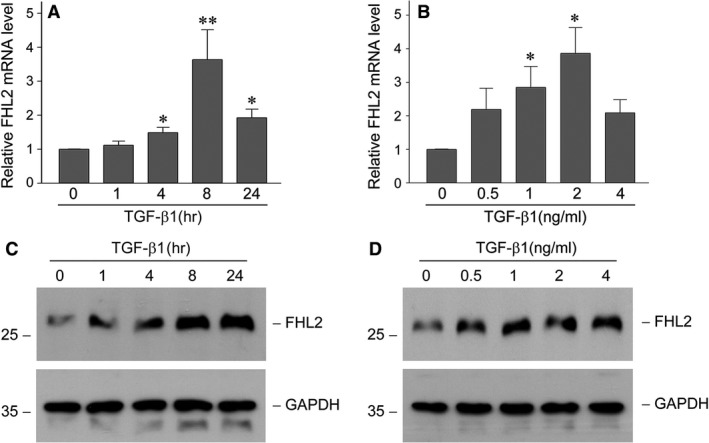
TGF‐β1 induces FHL2 mRNA and protein expression in tubular epithelial cells. (**A** and **B**) Quantitative real‐time RT‐PCR analyses display the induction of FHL2 mRNA by TGF‐β1. NRK‐52E cells were incubated with 2 ng/ml TGF‐β1 for various periods of time as indicated (**A**) or with various concentrations of TGF‐β1 for 8 hrs (**B**). *P <* *0.05, **P <* *0.01 *versus* without TGF‐β1 (*n* = 3). (**C** and **D**) Western blot analyses demonstrate that TGF‐β1 induced FHL2 protein expression in a time‐ and dosage‐dependent manner. NRK‐52E cells were treated with 2 ng/ml TGF‐β1 for various periods as indicated (**C**) or with various concentrations of TGF‐β1 for 24 hrs (**D**). Whole cell lysates were immunoblotted with specific antibodies against FHL2 and GAPDH, respectively.

We investigated FHL2 regulation by TGF‐β1 in NRK‐52E cells. Real‐time RT‐PCR analyses demonstrated that TGF‐β1 markedly induced FHL2 mRNA levels (Fig. 1A and B). Quantitative analyses revealed about 3.6‐fold induction of FHL2 mRNA at 8 hrs after 2 ng/ml TGF‐β1 treatment. FHL2 protein was also induced in a time‐ and dose‐dependent pattern by TGF‐β1, as confirmed by Western blot analyses (Fig. 1C and D).

**Figure 2:**
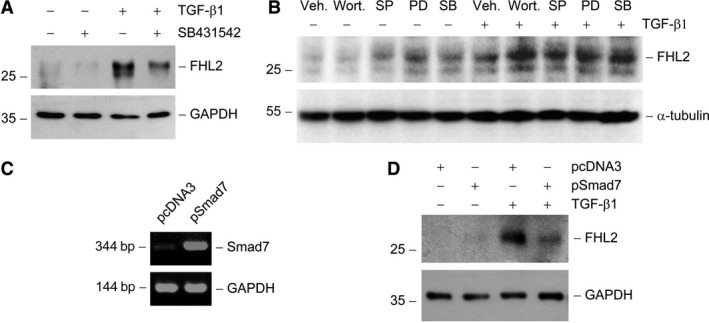
FHL2 induction by TGF‐β1 is dependent on intracellular Smad signalling. (**A**) TGF‐β receptor signalling is required for FHL2 induction. NRK‐52E cells were pre‐treated with activin receptor‐like kinase‐5 inhibitor SB431542 (10 μM), followed by incubation with TGF‐β1 (2 ng/ml) for 24 hrs. (**B**) Pharmacologic inhibition of different signal transduction pathways does not affect FHL2 induction by TGF‐β1. NRK‐52E cells were pre‐treated with either various chemical inhibitors or vehicle (Veh., 0.1% Me_2_ SO) for 30 min., followed by incubating in the absence or presence of 2 ng/ml TGF‐β1 for 24 hrs. Wort., wortmannin (PI3K inhibitor) (10 nM); PD, PD98059 (MEK1 inhibitor) (10 μM); SB, SB202190 (p38 MAPK inhibitor) (20 μM); SP, SP600125 (JNK inhibitor) (20 μM). (**C**) Semiquantitative RT‐PCR analyses confirm overexpression of Smad7 in NRK‐52E cells. NRK‐52E cells were transiently transfected with Smad7 expression vector (pSmad7) or empty vector (pcDNA3). (**D**) Western blot shows that overexpression of Smad7 suppressed FHL2 induction by TGF‐β1.

Figure 2A showed that TGF‐β1‐stimulated induction of FHL2 was largely abolished by TGF‐β type I receptor inhibitor 16, 28, which suggested that the induction of FHL2 is relied on TGF‐β receptor signalling. As is well known, TGF‐β1 could activate multiple intracellular signal pathways including Smad, p38 MAPK, ERK‐1/2, PI3K/Akt and JNK in tubular epithelial cells 16, 29, so we examined the potential signals that could take the responsibility for FHL2 induction by TGF‐β1. As shown in Figure 2B, impeding the activation of PI3K, p38 MAPK, ERK‐1/2 upstream kinase (MEK1) and JNK using specific chemical inhibitors did not abolish TGF‐β1‐mediated FHL2 induction. To elucidate the possible involvement of Smad signalling, we examined the effect of overexpression of Smad7, one of the inhibitory Smads 30, on FHL2 induction by TGF‐β1. Compared with empty vector transfection control, the ectopic expression of Smad7 almost completely abolished the TGF‐β1‐mediated FHL2 induction in NRK‐52E cells (Fig. 2D), suggesting that the induction of FHL2 by TGF‐β1 mainly count on Smad signalling.

### Ectopic expression of FHL2 induces tubular EMT

**Figure 3:**
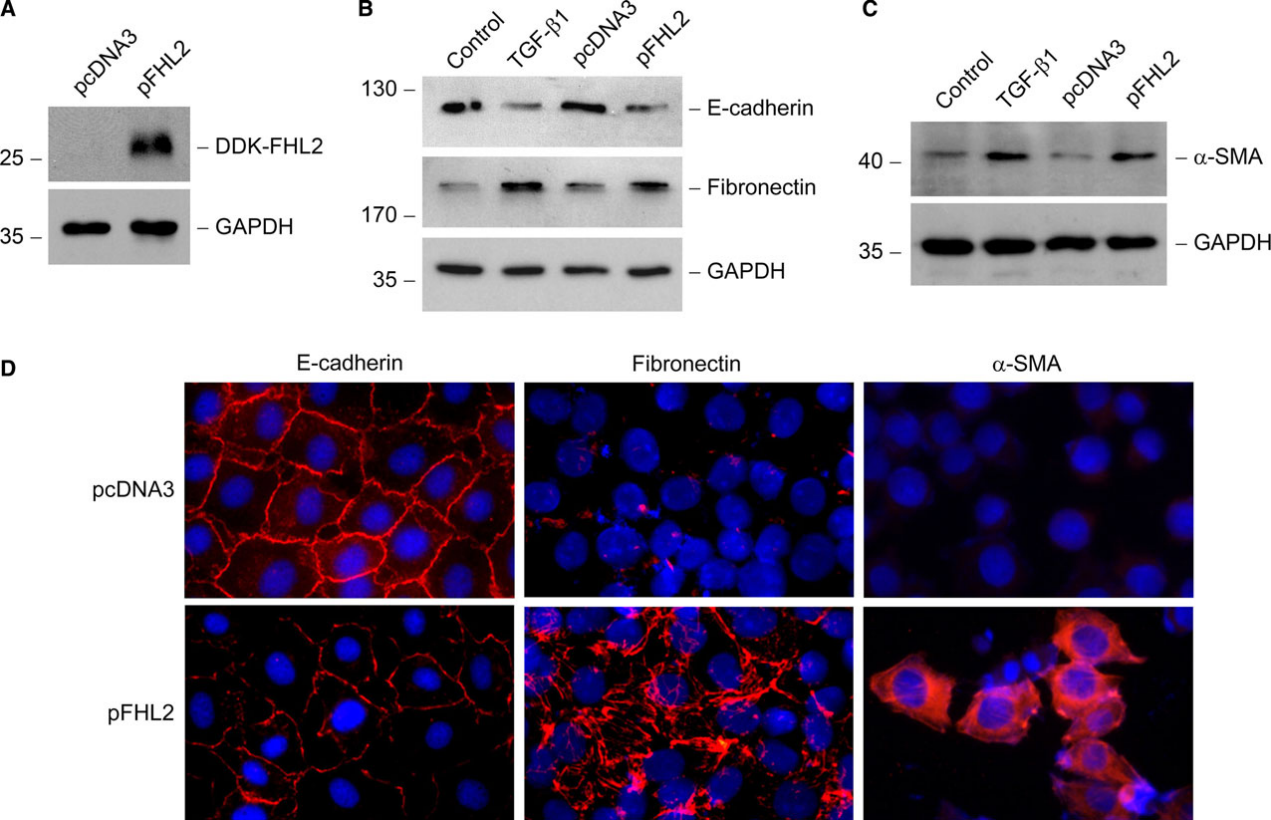
Ectopic expression of FHL2 suppresses E‐cadherin and induces α‐SMA and fibronectin. (**A**) Overexpression of FHL2 in tubular epithelial cells is confirmed by Western blotting with anti‐DDK antibody. NRK‐52E cells were transiently transfected with either the expression vector of DDK‐tagged FHL2 (pFHL2) or empty vector (pcDNA3). (**B** and **C**) Forced expression of FHL2 suppresses E‐cadherin expression (**B**) and induces fibronectin (**B**) and α‐SMA (**C**) expression and assembly in tubular epithelial cells. TGF‐β1 (2 ng/ml) treatment of NRK‐52E cells was used as positive control. (**D**) Immunofluorescence staining shows that overexpression of FHL2 suppresses epithelial markers E‐cadherin and induces fibronectin and α‐SMA expression in tubular epithelial cells.

For determining the function of FHL2 induction, FHL2 was overexpressed by transiently transfection with the expression vector of DDK‐tagged FHL2 in NRK‐52E cells. Ectopic expression of FHL2 was confirmed by Western blotting with anti‐DDK antibody (Fig. 3A). We found that overexpression of FHL2 inhibited expression of E‐cadherin, one epithelia marker, and induced expression of fibronectin, one main component of ECM (Fig. 3B). Overexpression of FHL2 also induced expression of α‐SMA, one marker of myofibroblasts (Fig. 3C). The dimension of E‐cadherin down‐regulation and fibronectin and α‐SMA up‐regulation incurred by ectopic expression of FHL2 was similar to that stimulated by TGF‐β1 (Fig. 3B and C). Immunofluorescence staining exhibited that overexpression of FHL2 led to a weakening of E‐cadherin expression in plasma membrane and a remarkable increase in fibronectin and α‐SMA expression and assembly (Fig. 3D). Therefore, these observations suggest that ectopic expression of FHL2 is able to trigger tubular EMT.

### Knockdown of FHL2 expression partially restrains TGF‐β1‐induced tubular EMT

**Figure 4:**
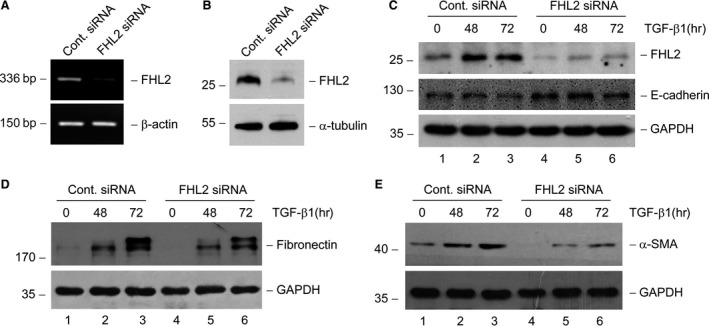
Knockdown of FHL2 expression partially restores E‐cadherin expression and inhibits α‐SMA and fibronectin induction by TGF‐β1. (**A** and **B**) Knockdown of endogenous FHL2 expression by small interfering RNA (siRNA) strategy is confirmed. RT‐PCR (**A**) and Western blot (**B**) demonstrate a reduced FHL2 mRNA and protein expression in NRK‐52E cells after transfection of FHL2‐specific siRNA. (**C**) Endogenous FHL2 expression is suppressed in the basal and TGF‐β1‐stimulated conditions, and knockdown of endogenous FHL2 partially restores E‐cadherin expression repressed by TGF‐β1. NRK‐52E cells were transfected with either control or FHL2 siRNA, followed by incubation with 2 ng/ml TGF‐β1 for various periods of time as indicated. (**D** and **E**) Knockdown of endogenous FHL2 suppresses fibronectin (**D**) and α‐SMA (**E**) expression in response to TGF‐β1 stimulation.

To further determine the potential role of FHL2 in tubular EMT, FHL2 expression was down‐regulated by transfection with specific FHL2 siRNA in NRK‐52E cells. Transfection with FHL2 siRNA not only led to a distinct decrease in endogenous FHL2 mRNA and protein levels (Fig. 4A and B), but also largely blocked FHL2 induction stimulated by TGF‐β1 (Fig. 4C). Knockdown of FHL2 expression appeared to up‐regulate E‐cadherin expression in the basal conditions (Fig. 4C, lane 4 *versus* lane 1) and partially restore E‐cadherin expression suppressed by TGF‐β1 (Fig. 4C, lane 5 *versus* lane 2, and lane 6 *versus* lane 3). In addition, down‐regulation of FHL2 partially prevented fibronectin and α‐SMA overproduction in response to TGF‐β1 stimulation (Fig. 4D and E, lane 5 *versus* lane 2, and lane 6 *versus* lane 3). Hence, it seems that knockdown of FHL2 expression partially inhibits tubular EMT induced by TGF‐β1.

### FHL2 is an endogenous regulator for β‐catenin signalling activation

**Figure 5:**
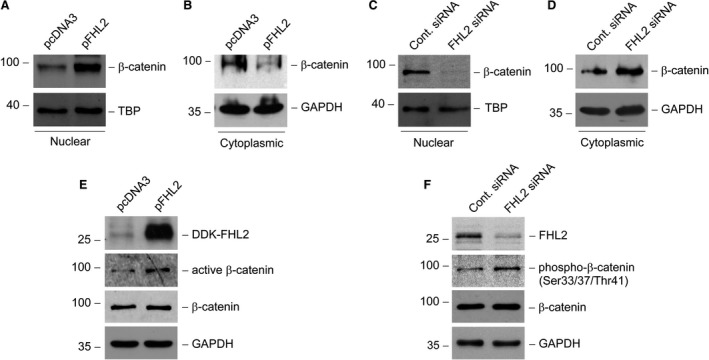
FHL2 is an endogenous regulator for β‐catenin nuclear translocation. (**A** and **B**) Ectopic expression of FHL2 promotes β‐catenin translocation into the nuclei. NRK‐52E cells were transiently transfected with either FHL2 expression vector (pFHL2) or empty vector (pcDNA3). (**C** and **D**) Knockdown of endogenous FHL2 promotes β‐catenin translocation out of the nuclei. NRK‐52E cells were transfected with either control or FHL2 siRNA. Nuclear and cytoplasmic proteins from whole cell lysates were prepared and immunoblotted with antibodies against β‐catenin, TBP or GAPDH, respectively (**A–D**). (**E**) Forced expression of FHL2 induces β‐catenin activation in tubular epithelial cells. NRK‐52E cells were transiently transfected with either pFHL2 or pcDNA3, and the whole cell lysates were immunoblotted with antibodies against DDK, active β‐catenin, β‐catenin or GAPDH, respectively. (**F**) Knockdown of endogenous FHL2 decreases β‐catenin activation. NRK‐52E cells were transfected with either control or FHL2 siRNA, and the whole cell lysates were immunoblotted with antibodies against FHL2, phosphorylated β‐catenin (Ser33/37/Thr41), β‐catenin or GAPDH, respectively.

Previous studies demonstrate that activation of β‐catenin signalling can induce tubular EMT 11 and that FHL2 is a coactivator of β‐catenin signalling in some other types of cell 22, 23, 31. To determine any possibility that β‐catenin is involved in the tubular EMT regulated by FHL2, we firstly investigated whether FHL2 affects the intracellular distribution of β‐catenin in tubular epithelial cells. For this purpose, we scrutinized β‐catenin subcellular localization after manipulating FHL2 expression in NRK‐52E cells. We found that forced expression of FHL2 induced β‐catenin nuclear translocation, whereas knockdown of endogenous FHL2 led to β‐catenin translocate out of the nuclei (Fig. 5A–D). The alteration of FHL2 abundance did not affect whole cellular β‐catenin level (Fig. 5E and F). As it is the dephosphorylation and stabilization of β‐catenin that allow it to shift into the nucleus, we next investigated whether the changed amount of FHL2 has an influence on the phosphorylation of β‐catenin. We found that overexpression of FHL2 resulted in an increase in dephosphorylated (Ser37/Thr41), active β‐catenin level in NRK‐52E cells (Fig. 5E). Conversely, down‐regulation of endogenous FHL2 induced an increase in phosphorylated (Ser33/37/Thr41) β‐catenin level (Fig. 5F). These results suggest that FHL2 is a regulatory factor for the stabilization and nuclear translocation of β‐catenin *via* affecting its phosphorylation. Given that the nuclear translocation of β‐catenin is an indispensable procedure for it to control its target genes transcription in nucleus, FHL2 could be an endogenous mediator for modulating the activity of β‐catenin signalling during the process of tubular EMT.

### FHL2 regulates β‐catenin‐mediated gene transcription

**Figure 6:**
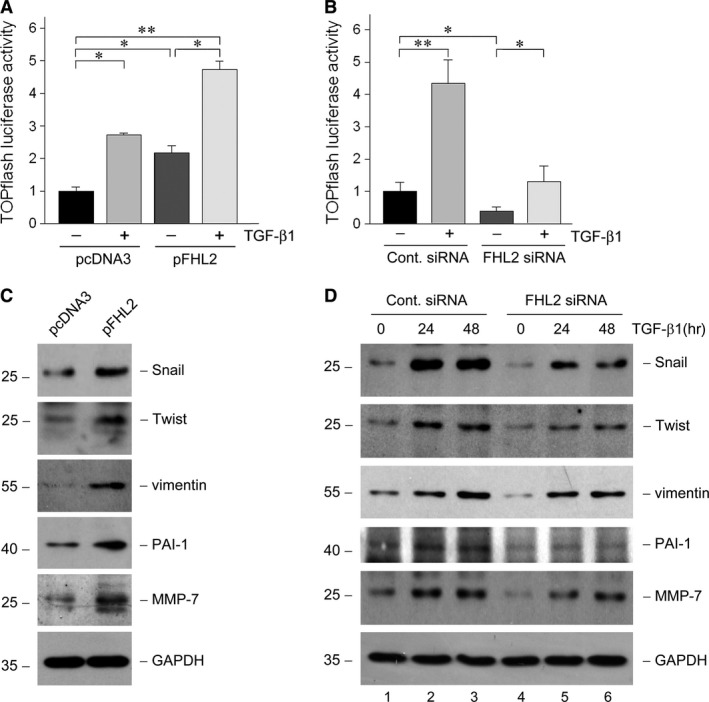
FHL2 regulates β‐catenin‐mediated gene transcription. (**A**) NRK‐52E cells were cotransfected with TOPflash reporter plasmid and either pcDNA3 or pFHL2 vector, followed by incubation with or without 2 ng/ml TGF‐β1. Relative luciferase is reported (*n *= 3). *P <* *0.05, **P <* *0.01. (**B**) NRK‐52E cells were cotransfected with TOPflash reporter plasmid and either control or FHL2 siRNA, followed by incubation with or without 2 ng/ml TGF‐β1. Relative luciferase is reported (*n *= 3). *P <* *0.05, **P <* *0.01. (**C**) Ectopic expression of FHL2 promotes the expression of β‐catenin target genes. NRK‐52E cells were transiently transfected with either pcDNA3 or pFHL2 vector, and the cell lysates were immunoblotted with antibodies against Snail, Twist, vimentin, PAI‐1, MMP‐7 or GAPDH, respectively. (**D**) Knockdown of endogenous FHL2 expression partially inhibits β‐catenin target genes induction stimulated by TGF‐β1. NRK‐52E cells were transfected with either control or FHL2 siRNA, followed by incubation with 2 ng/ml TGF‐β1 for 24 or 48 hrs, and the cell lysates were immunoblotted with various antibodies as indicated.

For assessing the functional consequence of FHL2‐regulated β‐catenin signalling activation in tubular epithelial cells, we examined the β‐catenin‐mediated transcriptional activity in a TOPflash luciferase reporter system. We found that either exogenous FHL2 or TGF‐β1 markedly induced β‐catenin‐mediated transcriptional activity in NRK‐52E cells (Fig. 6A). A combination of overexpression FHL2 and TGF‐β1 stimulation further increased the transcriptional activity (Fig. 6A). To confirm the importance and necessity of FHL2 in regulating β‐catenin‐mediated gene transcription, we utilized the specific siRNA to down‐regulate FHL2 expression and found that knockdown of FHL2 not only inhibited β‐catenin‐mediated transcriptional activity at basal condition, but also restrained the transcriptional activity stimulated by TGF‐β1 (Fig. 6B).

Given that the activation of β‐catenin signalling plays a crucial role in mediating tubular EMT, we further examined the effect of FHL2 on the expression of β‐catenin target, EMT‐related genes, including Snail, Twist, vimentin, PAI‐1 and MMP‐7. We found that forced expression of FHL2 was able to notably induce these genes expression in NRK‐52E cells (Fig. 6C). Conversely, knockdown of FHL2 seemed to attenuate these genes expression at basal condition (Fig. 6D, lane 4 *versus* lane 1) and hinder their induction stimulated by TGF‐β1 to varying degrees (Fig. 6D, lane 5 *versus* lane 2, and lane 6 *versus* lane 3). Therefore, it is suggested that through regulating β‐catenin‐mediated transcriptional activity and β‐catenin‐targeted gene expression, FHL2 plays an essential role in governing tubular EMT.

### FHL2/β‐catenin interaction is induced in TGF‐β1‐stimulated tubular epithelial cells

**Figure 7:**
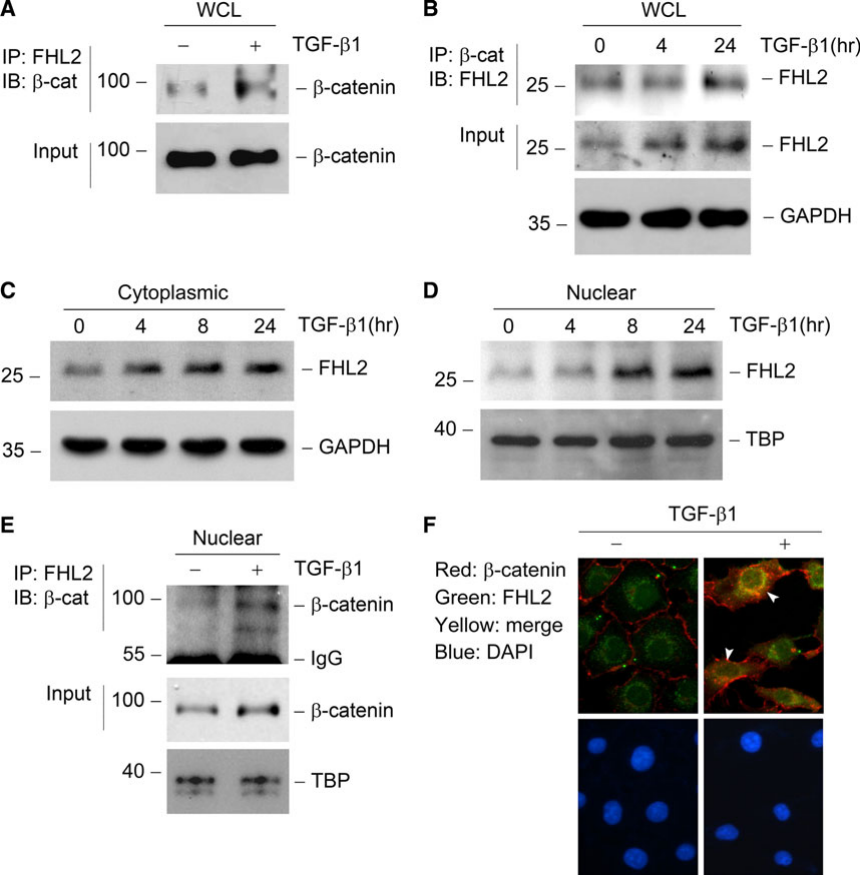
TGF‐β1 induces FHL2 to interact with β‐catenin, especially in the nuclei. (**A** and **B**) Coimmunoprecipitation (IP) reveal that TGF‐β1 induces FHL2/β‐catenin complex formation in tubular epithelial cells. Cell lysates were prepared after TGF‐β1 treatment as indicated and immunoprecipitated with specific antibody against FHL2 or β‐catenin, followed by immunoblotting for β‐catenin (**A**) or FHL2 (**B**), respectively. Cellular β‐catenin (**A**) or FHL2 and GAPDH (**B**) levels were assessed by routine Western blot analysis of whole cell lysate (WCL), respectively. (**C** and **D**) TGF‐β1 induces FHL2 expression both in the cytosol (**C**) and nuclei (**D**) in tubular epithelial cells. NRK‐52E cells were treated with 2 ng/ml TGF‐β1 for various periods as indicated, and the nuclear and cytoplasmic proteins from WCL were prepared and immunoblotted with antibodies against FHL2, TBP or GAPDH, respectively. (**E**) IP demonstrates a FHL2/β‐catenin complex formation induced by TGF‐β1 in the nuclei of tubular epithelial cells. The nuclear protein from WCL was prepared and immunoprecipitated with specific antibody against FHL2, followed by immunoblotting for β‐catenin. Nuclear β‐catenin and TBP levels were assessed by routine Western blot analysis of nuclear protein, respectively. (**F**) Immunofluorescence staining exhibits the colocalization of FHL2 and β‐catenin in tubular epithelial cells. NRK‐52E cells were treated with 2 ng/ml TGF‐β1 for 24 hrs, followed by staining with anti‐FHL2 and anti‐β‐catenin antibodies. Arrowheads indicate the colocalization sites in NRK‐52E cells. DAPI, 4′, 6‐diamidino‐2‐phenylindole‐HCl.

For exploring the potential reason that FHL2 affects β‐catenin signalling in tubular epithelial cells, we examined the interaction of FHL2 with β‐catenin under TGF‐β1‐stimulated condition using a coimmunoprecipitation approach. Incubation of NRK‐52E cells with TGF‐β1 induced FHL2 to interact with β‐catenin, as depicted by increased FHL2/β‐catenin complex formation after TGF‐β1 treatment (Fig. 7A and B). Using a cytoplasmic and nuclear protein extraction assay, we found that both cytoplasmic and nuclear FHL2 levels were markedly increased in a time‐dependent pattern in TGF‐β1‐treated NRK‐52E cells (Fig. 7C and D), and the amount of β‐catenin in the nuclei was increased too (Fig. 7E). As both FHL2 and β‐catenin go through nuclear translocation after TGF‐β1 treatment, we further investigated whether FHL2 interacts with β‐catenin in the nuclei. We found that the FHL2/β‐catenin complex formation was increased in the nuclear protein extracted from cells incubated with TGF‐β1 (Fig. 7E). Immunofluorescence staining displayed that TGF‐β1 treatment caused colocalization of FHL2 and β‐catenin in NRK‐52E cells, especially in the nuclei (Fig. 7F, arrowhead), while at basal conditions, FHL2 was weakly diffused in cytoplasm with partial denseness around the perinuclear space and β‐catenin exhibited a plasma membrane staining pattern (Fig. 7F). Thus, following the induction of FHL2, the interaction of FHL2 with β‐catenin is increased after TGF‐β1 stimulation.

### Renal β‐catenin activity is correlated with FHL2 induction in obstructive nephropathy

**Figure 8:**
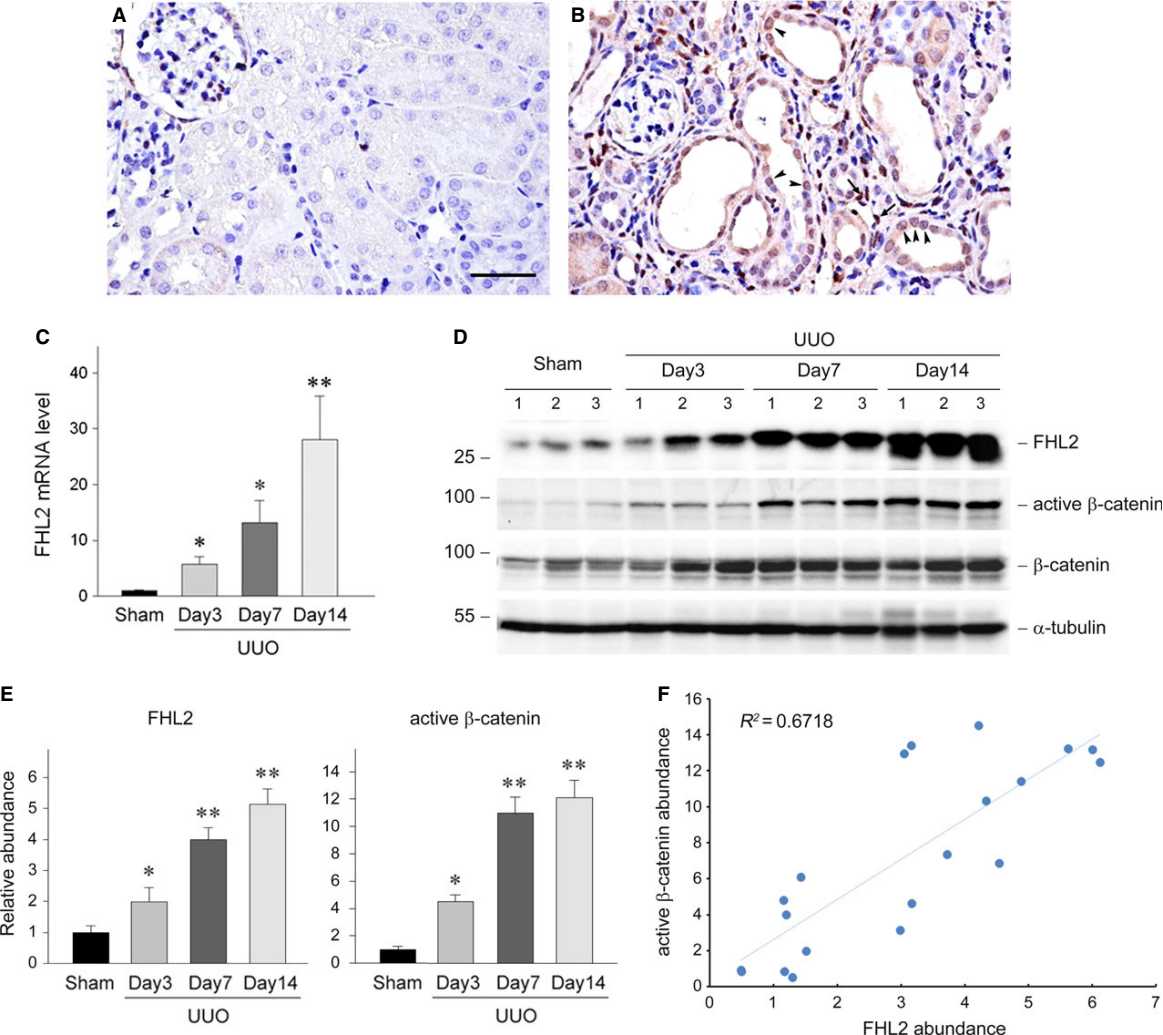
Renal β‐catenin activity is closely correlated with FHL2 induction in a mouse model of obstructive nephropathy. (**A** and **B**) Representative micrographs display the induction and localization of FHL2 in fibrotic kidney. Kidneys from sham (**A**) and UUO for 7 days (**B**) were stained immunohistochemically for FHL2 protein. Bar = 40 μm. Arrowheads and arrows indicate FHL2‐positive tubular epithelial cells and FHL2‐positive cells in the interstitium, respectively. (**C**) Quantitative real‐time RT‐PCR data on renal FHL2 mRNA level in different groups of mice as indicated. *P <* *0.05, **P <* *0.01 *versus* sham controls (*n *=* *4). (**D** and **E**) Representative Western blot analyses (**D**) and quantitative data (**E**) show the induction of renal FHL2 protein and the activation of β‐catenin at different time‐points after UUO. Numbers (1, 2 and 3) in D indicate each individual animal in a given group. *P <* *0.05, **P <* *0.001 *versus* sham controls (*n *=* *5). (**F**) Linear regression shows a close correlation between renal active β‐catenin abundance and FHL2 abundance (arbitrary units). The correlation coefficient (*R* ^2^) is shown.

For detecting the potential role of FHL2 in regulating tubular EMT *in vivo*, we investigated FHL2 expression in obstructive nephropathy, a mouse model of renal interstitial fibrosis. Immunohistochemical staining exhibited that compared with the sham controls (Fig. 8A), FHL2 protein was markedly up‐regulated and partially localized in tubular epithelial cells, especially in the nuclei (Fig. 8B, arrowheads), in the fibrotic kidney at day 7 after UUO (Fig. 8B). Of note, quite a lot of FHL2‐positive cells were also observed in the interstitium (Fig. 8B, arrows). Real‐time RT‐PCR and Western blot analyses demonstrated that FHL2 mRNA (Fig. 8C) and protein (Fig. 8D and E) levels were remarkably increased in a time‐dependent pattern in the obstructed kidney. The significant induction of FHL2 mRNA and protein were observed as early as day 3 after UUO, which is the time‐point before the initiation of tubular EMT in this model 32. These observations suggest that the induction of FHL2 may participate in regulating tubular EMT *in vivo*.

To further uncover the correlation between FHL2 induction and Wnt/β‐catenin signalling activation *in vivo*, we examined the levels of dephosphorylated (Ser37/Thr41), active β‐catenin and β‐catenin. Expectedly, Wnt/β‐catenin signalling was dramatically activated, as illustrated by a time‐dependent increase in active β‐catenin level in the obstructed kidneys (Fig. 8D and E). Renal β‐catenin protein also significantly induced in a time‐dependent manner in this model (Fig. 8D), as previously reported 18. Linear regression displayed a close correlation between renal active β‐catenin abundance and FHL2 abundance (Fig. 8F), suggesting that the induction of FHL2 may promote tubular EMT, at least partially, *via* regulating β‐catenin activity in this model.

## Discussion

Our findings presented in current study suggest that FHL2, an adaptor protein containing LIM domains only, through modulating β‐catenin signalling, implicates in the regulation of TGF‐β1‐induced tubular EMT. Our data demonstrate that FHL2 expression is induced by TGF‐β1, which largely relies on intracellular Smad signalling, a crucial pathway for mediating tubular EMT. Furthermore, ectopic expression of FHL2 leads to repression of E‐cadherin and induction of fibronectin and α‐SMA, an effect that is similar to that by TGF‐β1. Conversely, knockdown of FHL2 partially restores E‐cadherin and ameliorates the induction of fibronectin and α‐SMA stimulated by TGF‐β1. In addition, TGF‐β1 causes increased FHL2 abundance both in cytosol and nuclei and an increase in the interaction between FHL2 and β‐catenin in tubular epithelial cells, especially in the nuclei. It seems that the increased FHL2 abundance by TGF‐β1 brings a change of β‐catenin phosphorylation and subcellular localization, which results in activation of β‐catenin signalling, exhibiting the enhancement of β‐catenin transcriptional activity and its target genes expression. In a mouse model of obstructive nephropathy, FHL2 expression is induced and the activation of β‐catenin signalling is closely correlated with FHL2 induction. These results have established that FHL2, acting as an endogenous regulator for β‐catenin signalling activity, implicates in the regulation of tubular EMT, thereby participating in the pathogenesis of renal fibrosis.

Belonging to LIM domain‐only protein family, FHL protein possesses four complete and one half LIM homeodomains. FHL2 is the most studied one among five members of FHL protein subfamily, that is FHL1, FHL2, FHL3, FHL4 and ACT. As LIM domain serves as a specific scaffold, protein containing LIM domains exerts its functions through interacting with various proteinaceous binding partners 19, 20, 21. FHL2 exhibits specific interactions with a number of proteins, including transcription factors and coregulators, transmembrane receptors, structural proteins and enzymes. Through regulating the function of these proteins, FHL2 plays a role in modulating multiple intracellular signal pathways involved in multifarious cellular processes 22. It has been indicated that β‐catenin is one of protein partners for FHL2, and *via* interacting with β‐catenin, FHL2 could affect β‐catenin‐mediated gene transcription in a cell‐specific manner 19. In current study, the increased abundance of FHL2 leads to β‐catenin translocation into the nuclei and the activation of this signalling in tubular epithelial cells. This observation is in accord with several previous studies presenting that FHL2 facilitates the formation of a FHL2–β‐catenin protein complex in the nucleus where FHL2 serves as a coregulator for β‐catenin‐mediated transcription 31, 33. It should be pointed out that FHL2 could be a coactivator or corepressor of β‐catenin depending on cell types 19. In one recent study, FHL2 presents as a coactivator of Wnt/β‐catenin signalling in podocytes in DKD 23. Consistently, our data suggest that FHL2 has both physical and functional interactions with β‐catenin in the tubular epithelial cells undergoing EMT.

FHL2 is predominantly expressed in muscle and cardiovascular systems, and it can also be found in tissues of different origin 20. Recent study shows that FHL2 expression is abundant in podocytes in adult kidney tissue, and its expression is up‐regulated by high glucose and cytokines associated with DKD, such as angiotensin II and TGF‐β1 23. However, it is barely reported regarding its expression and function in tubular epithelial cells, especially in certain pathologic states, for example tubular EMT. The current study presents that FHL2 expression is induced at both mRNA and protein levels during tubular EMT stimulated by TGF‐β1. It seems that the induction of FHL2 by TGF‐β1 happens at the gene transcription stage because FHL2 mRNA level is markedly increased after TGF‐β1 treatment. In addition, the inhibition of Smad signalling by overexpressing Smad7 diminishes the induction of FHL2 by TGF‐β1, so it is a plausible deduction that FHL2 could be a downstream target of TGF‐β1/Smad signalling during tubular EMT.

One of the significative findings in the present study is that the role of FHL2 involved in TGF‐β1‐induced tubular EMT is dependent on, at least partially, its modulation for the activity of β‐catenin signalling. Observations suggesting this notion are as follows. First, after TGF‐β1 treatment, the abundance of FHL2 is increased both in cytosol and nucleus, and FHL2 physically interacts with β‐catenin, especially in the nuclei. Second, ectopic expression of FHL2 causes activation of β‐catenin signalling, whereas knockdown of endogenous FHL2 leads to inhibition of β‐catenin signalling activity. Third, after short‐term TGF‐β1 stimulation, the abundance of β‐catenin translocated into nucleus, β‐catenin transcriptional activity and its target genes expression are all increased, while the total β‐catenin abundance is not changed. However, down‐regulation of FHL2 attenuates β‐catenin transcriptional activity and its target genes expression evoked by TGF‐β1. Therefore, it is conceivable to postulate that TGF‐β1‐induced up‐regulation of FHL2 facilitates tubular EMT substantially through binding to β‐catenin, which results in the enhancement of β‐catenin transcriptional activity in the nuclei.

The authentic role of tubular EMT contributing to the pathogenesis of renal interstitial fibrosis has been debated for several years. Studies using genetic cell lineage tracing indicate that the major portion of myofibroblasts having the ability to produce ECM in renal interstitium does not originate from tubular EMT 3, 5. However, based on recent studies, tubular epithelial cells go through a partial EMT after injury, resulting in cell cycle arrest, crippled tubular regeneration and interstitial fibroblasts activation. These reports suggest that EMT is a central process in the cycle of tubular injury and repair and thus augments tissue damage and renal fibrosis. Hence, hindering tubular EMT could break the vicious cycle and impede the development of renal fibrosis 8, 9, 10. Signal pathways such as TGF‐β1/Smad and Wnt/β‐catenin play important roles in the regulation of tubular EMT and have been proposed as possible intervention targets for renal fibrosis 11, 34, 35. In the present study, TGF‐β1 induces FHL2 expression and the up‐regulation of FHL2 activates β‐catenin signalling, whereas down‐regulation of FHL2 attenuates β‐catenin signalling activity induced by TGF‐β1. Therefore, FHL2 provides a novel regulatory link bridging TGF‐β1 and β‐catenin signalling during the process of tubular EMT. One previous report identifies Snail1 as a binding protein of FHL2 and suggests that by interacting with Snail1, FHL2 inhibits E‐cadherin transcription activity, which promotes the EMT process in colon cancer cells 36. This regulatory mechanism remains to be clarified in the process of tubular EMT. Although it is possible that FHL2 may promote tubular EMT through other mechanisms or that TGF‐β1 may activate β‐catenin signalling through other pathways, our results demonstrate that FHL2 is an endogenous regulator for β‐catenin signalling and thus implicates in the modulation of TGF‐β1‐induced tubular EMT.

For all we know, this is the first report displaying that FHL2 is up‐regulated in fibrotic kidney. The concomitant of β‐catenin activation with FHL2 induction in obstructive nephropathy (Fig. 8) suggests that FHL2 may participate in mediating tubular EMT through regulating β‐catenin activity in this model. Our findings imply that it is worthwhile to further investigate the regulation and function of FHL2 and the interaction between FHL2 and β‐catenin during tubular EMT *in vivo*.

In summary, we have presented herein that the induction of FHL2 by TGF‐β1 plays a crucial role for β‐catenin signalling activation and tubular EMT progression. In addition, our studies indicate that through interaction with β‐catenin, FHL2 acts as a pivotal mediator for TGF‐β1 regulating β‐catenin signalling in tubular epithelial cells. To be sure, further investigations on the regulation and function of FHL2–β‐catenin interaction in the circumstance of renal fibrosis *in vivo* will be helpful to bring new insights into the mechanism of tubular EMT.

## Conflict of interest

The authors confirm that there is no conflict of interests.
